# In vivo analysis of fracture toughness of thyroid gland tumors

**DOI:** 10.1186/1754-1611-2-12

**Published:** 2008-10-06

**Authors:** Nagesh Ragavendra, JW Ju, James W Sayre, Sharon Hirschowitz, Inder Chopra, Michael W Yeh

**Affiliations:** 1 Department of Radiology, The David Geffen School of Medicine at UCLA, Box 957437, 757 Westwood Plaza, Los Angeles, CA 90095, USA; 2 Department of Civil & Environmental Engineering, UCLA, 10833 LeConte Avenue, Los Angeles, CA 90095, USA; 3 Pathology, The David Geffen School of Medicine at UCLA, Box 957437, 757 Westwood Plaza, Los Angeles, CA 90095, USA; 4 Medicine, The David Geffen School of Medicine at UCLA, Box 957437, 757 Westwood Plaza, Los Angeles, CA 90095, USA; 5 Surgery, The David Geffen School of Medicine at UCLA, Box 957437, 757 Westwood Plaza, Los Angeles, CA 90095, USA; 6 Biostatistics The David Geffen School of Medicine at UCLA, Box 957437, 757 Westwood Plaza, Los Angeles, CA 90095, USA

## Abstract

**Background:**

Human solid tumors that are hard or firm on physical palpation are likely to be cancerous, a clinical maxim that has been successfully applied to cancer screening programs, such as breast self-examination. However, the biological relevance or prognostic significance of tumor hardness remains poorly understood. Here we present a fracture mechanics based *in vivo *approach for characterizing the fracture toughness of biological tissue of human thyroid gland tumors.

**Methods:**

In a prospective study, 609 solid thyroid gland tumors were percutaneously probed using standard 25 gauge fine needles, their tissue toughness ranked on the basis of the nature and strength of the haptic force feedback cues, and subjected to standard fine needle biopsy. The tumors' toughness rankings and final cytological diagnoses were combined and analyzed. The interpreting cytopathologist was blinded to the tumors' toughness rankings.

**Results:**

Our data showed that cancerous and noncancerous tumors displayed remarkable haptically distinguishable differences in their material toughness.

**Conclusion:**

The qualitative method described here, though subject to some operator bias, identifies a previously unreported *in vivo *approach to classify fracture toughness of a solid tumor that can be correlated with malignancy, and paves the way for the development of a mechanical device that can accurately *quantify *the tissue toughness of a human tumor.

## Background

Human solid cancers typically are harder and firmer than surrounding normal tissue upon clinical palpation [1]. This characteristic has been linked to the presence of abundant collagen in the tumor stroma, commonly referred to as the desmoplastic reaction [2]. While the origin of cancer is genetically based, accumulating evidence now indicates that the tumor-associated desmoplastic stroma is critical to cancer's growth and progression [3]. For example, *in vitro *studies have shown that stromal rigidity or stiffness strongly influences normal cell motility and tissue form, and may even select a malignant phenotype [4,5]. There is growing realization that therapeutic intervention aimed at normalizing the tumor's stromal compartment may halt or even reverse the course of cancer growth [3]. Thus, an understanding of the interaction between tumor cells and their mechanical microenvironment is seen as increasingly relevant to the study of tumor biology. However, how best to measure, interpret, and model the mechanical attributes of tumor stromal tissue *in vivo *remain unclear. A novel technique, termed elastography [6-8], has shown promise in this regard and involves *in vivo *assessment of local responses of the tissue to an applied load (i.e., mechanical indentation) by imaging the longitudinal or shear-strain components at different locations in the tissue. In the present study, we have taken a different approach to this problem and report a fracture mechanics based *in vivo *method for testing the fracture toughness of solid thyroid gland tumors by manual probing using hypodermic needles.

During routine sonographically-guided fine needle biopsy of solid tumors [nodules] of the human thyroid gland, it was serendipitously observed by one of the authors (NR) that the nature and strength of the haptic force feedback cues varied among tumors. To further investigate this phenomenon and to assess the relationship between tumor hardness and cytological diagnosis, a prospective clinical study was designed and implemented incorporating the principles of fracture mechanics [9] with haptic modality [10].

Fracture mechanics governs how materials [solid and semisolid] behave before and after the nucleation and growth of micro and macro-cracks, and describes how cracks initiate and propagate in materials. Fracture toughness characterizes the material's intrinsic penetration resistance [fracture energy] to crack initiation and propagation. In the present study, the manually instigated thrusting movement of the probing needle provided the necessary force for the initiation and propagation of fractures through solid tumor tissue. However, what causes the build-up of intratumoral resistance to needle penetration is unclear. Recent data [11] show that in addition to abundant collagen deposition, structural realignment of collagen fibers, especially around groups of proliferating cancer cells, is a prominent feature within the growing tumor. We hypothesize that as the needle tip penetrates the tumor core, irreversible mechanical damage to the collagen tissue ensues which results in rapid disassociation of collagen fibers into subfibers, fibrils and microfibrils. The fracture energy that is released during this process is potentially quantifiable and may signify a unique mechanical tumor marker.

In the present investigation, we have analyzed the nature of the penetration resistance (fracture energy) *qualitatively *by means of extended haptic perception [12]. Haptic perception entails an active exploration of an object over time and space by integrating the body's sensory and kinesthetic abilities. This is known as active touch [10]. Since it is dependent upon the exchange of mechanical force cues, a man-made inorganic tool, such as a blind person's cane or a dental probe, can effectively extend the perception beyond the body's physical boundary [12]. In the present study, the fine needle used as an embodiment of haptic tool effectively extended the perception from the fingertip to the needle tip.

## Results

Table 1 shows the distribution of cancer among 609 thyroid tumors categorized into Groups 1 and 2 on the basis of the nature and strength of haptic force cues. Bayesian analysis of data [Table 2] shows dramatic differences in the material toughness between cancerous and noncancerous tumors; 64% of Group 1 tumors exhibited thyroid cancer, while 95% of Group 2 tumors displayed no histological evidence of cancer.

**Table 1:** Thyroid cancer distribution in patient Groups 1 and 2 [N = 609]

	Group 1	Group 2
Cancer Present	93	22
Cancer Absent	53	441

**Table 2:** Bayesian analysis of data. [N = 609]

Sensitivity	0.81
Specificity	0.89
Positive Predictive Value	0.64
Negative Predictive Value	0.95
Positive Likelihood Ratio	7.54
Negative Likelihood Ratio	0.21
Negative Post-Test Odds	0.05
Positive Post-Test Odds	1.75
Odds Ratio	35.17
False Positive Rate	0.11
False Negative Rate	0.19

## Discussion

The results described in this report, though subject to some operator bias, demonstrate the feasibility of an *in vivo *approach for characterizing the fracture toughness of a solid tumor that can be correlated with malignancy. However, these results need to be validated and verified by *quantitative *analysis. We anticipate our method to be a good starting point for the development of a mechanical device that can *quantify *the tumor's toughness *in vivo *using the fracture mechanics principles and fracture toughness measurements.

## Conclusion

A qualitative *in vivo *method for characterizing a solid tumor's fracture toughness by haptic means is reported. These results when validated by *quantitative *measurement data are likely to provide a new framework for understanding the mechanical attributes of tumor microenvironment.

## Methods

**Figure 1:**
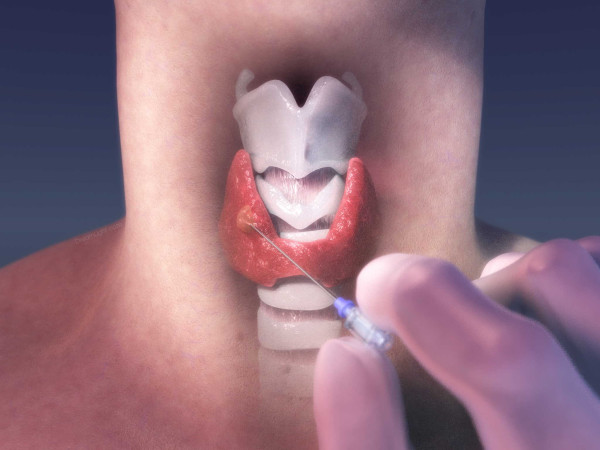
**A 3D rendered image of a right thyroid lobe solid tumor about to be pierced and probed manually with a fine needle.** Haptic force feedback cues generated by the probing needle were used to characterize and classify a solid tumor into two groups. Tumors exhibiting haptic cues that evoked the perception of cutting through an unripe pear [hard and gritty] were placed in Group 1, while those with the perception of cutting through jelly [soft and spongy] in Group 2. The results were dramatic: 64% of Group 1 tumors were cancerous while 95% of Group 2 tumors were noncancerous.

Solid tumors of the human thyroid gland [Fig. 1] were selected for testing because of their ready anatomic accessibility for direct clinical intervention; i.e., via fine-needle biopsy, and their high clinical prevalence in the United States population. The lead author (NR) was solely responsible for ranking the tissue hardness of all 609 thyroid gland tumors and subsequent fine needle biopsy. The tissue samples were processed on-site by a cytotechnologist and hand delivered to the cytopathologist for interpretation and final diagnosis. The interpreting cytopathologist was blinded to the tumors' hardness rankings.

The method, monitored by real-time sonographic image guidance [see Additional file 1] is described as follows: [a] after percutaneous introduction, the 5-cm long, 25-gauge fine needle is advanced through soft tissues of the neck, and its tip inserted into the tumor surface, [b] a fracture is initiated by the forward movement of the needle tip, [c] the fracture is propagated through the solid core by repeatedly advancing and withdrawing the needle, and [d] the tumor hardness is ranked on the basis of the nature and strength of the haptic force-feedback cues emanating from within the tumor due to tissue penetration and rupture induced by the rapidly moving needle tip.

Ranked tumors were placed in one of two groups [Table 1]: Group 1 [N = 134]: tumors exhibiting penetration resistance with a distinctive force-feedback cue, as if cutting through an unripe pear; Group 2 [N = 475]: tumors exhibiting no resistance, as if cutting through jelly. The toughness rankings were then correlated to the final cytological diagnoses.

## Competing interests

The authors declare that they have no competing interests.

## Authors' contributions

NR conceived of the study; substantially contributed to its design, acquisition and interpretation of data; wrote the paper and approved the overall manuscript. JWJ participated in revising the manuscript critically for important intellectual content. JWS performed the statistical analysis. SH carried out the cytological studies and interpreted the data in a double blind fashion. IC and MWY participated in drafting the manuscript. All authors read and approved the final manuscript.

## Supplementary Material

Additional file 1 This movie clip demonstrates the technique of manual probing of the solid thyroid tumor with a fine needle. Note the apparent ease with which the needle travels through a non-cancerous tumor. In contrast, a cancerous tumor offers substantial resistance to needle insertion and penetration. Remarkable differences in haptic force cues were apparent between cancerous and benign tumors. Click here for file
